# Congenital subependymal giant cell astrocytomas in patients with tuberous sclerosis complex

**DOI:** 10.1007/s00381-014-2555-8

**Published:** 2014-09-17

**Authors:** Katarzyna Kotulska, Julita Borkowska, Marek Mandera, Marcin Roszkowski, Elzbieta Jurkiewicz, Wiesława Grajkowska, Małgorzata Bilska, Sergiusz Jóźwiak

**Affiliations:** 1 Department of Neurology and Epileptology, The Children’s Memorial Health Institute, Warsaw, Poland; 2 Department of Science, Department of Neurology and Epileptology, The Children’s Memorial Health Institute, Al. Dzieci Polskich 20, 04-730 Warsaw, Poland; 3 Department of Paediatric Neurosurgery, Silesian Medical University, Katowice, Poland; 4 Department of Neurosurgery, The Children’s Memorial Health Institute, Warsaw, Poland; 5 Department of Radiology, The Children’s Memorial Health Institute, Warsaw, Poland; 6 Department of Pathology, The Children’s Memorial Health Institute, Warsaw, Poland

**Keywords:** Subependymal giant cell astrocytoma, Tuberous sclerosis complex, Infant, Newborn

## Abstract

**Purpose:**

Subependymal giant cell astrocytoma (SEGA) is a brain tumor associated with tuberous sclerosis complex (TSC). It usually grows in a second decade of life, but may develop in the first months of life. The aim of this work was to establish the incidence, clinical features, and outcome of congenital SEGA in TSC patients.

**Methods:**

Cohort of 452 TSC patients was reviewed to identify cases with growing or hydrocephalus producing SEGAs in the first 3 months of life. Clinical presentation, size of the tumor, growth rate, mutational analysis, treatment applied, and outcome were analyzed.

**Results:**

Ten (2.2 %) patients presented with SEGA in the first 3 months of life. All of them had documented SEGA growth and all developed hydrocephalus. In eight patients, mutational analysis was done, and in all of them, TSC2 gene mutations were identified. Mean maximum SEGA diameter at baseline was 21.8 mm. Mean SEGA growth rate observed postnatally was 2.78 mm per month and tended to be higher (5.43 mm per month) in patients with TSC2/PKD1 mutation than in other cases. Seven patients underwent SEGA surgery and surgery-related complications were observed in 57.1 % cases. One patient was successfully treated with everolimus as a primary treatment.

**Conclusions:**

Congenital SEGA develops 2.2 % of TSC patients. Patients with TSC2 mutations, and especially with TSC2/PKD1 mutations, are more prone to develop SEGA earlier in childhood and should be screened for SEGA from birth. In young infants with SEGA, both surgery and mTOR inhibitor should be considered as a treatment option.

## Introduction

Subependymal giant cell astrocytoma (SEGA) is a rare low-grade brain tumor associated almost exclusively with tuberous sclerosis complex (TSC) [5, 10, 11]. TSC is a genetically determined disorder that affects approximately 1 child in 6000 [28]. It is characterized by the development of benign tumors in various tissues and organs and brain lesions, including SEGAs, subependymal nodules (SENs), and cortical tubers as well as epilepsy and its comorbidities, present the major cause of mortality and morbidity in affected individuals [11, 35]. In TSC, inactivating mutations in either of two genes, *TSC1* or *TSC2*, lead to hyperactivation of mammalian target of rapamycin (mTOR) pathway, which is considered to be a hallmark of the disease [15, 19]. Mutations in *TSC2* were reported to generate a more severe phenotype than mutations in *TSC1* [6].

Usually, SEGAs grow in children and adolescents, but there are case reports on neonatal presentation of SEGAs in TSC patients [1, 13, 22, 24, 25, 27, 31–33, 36]. However, there are no data on the incidence of SEGAs in newborns and small infants with TSC and the treatment recommendations for that age group of patients. Currently, there are two possible treatment options for SEGA: surgery or mTOR inhibitor, everolimus, which has been approved for SEGA associated with TSC by FDA and EMA [3, 14]. The data on safety and efficacy of any of these treatments in newborns and infants with SEGA are very limited [7, 18, 20, 25, 32].

The aim of this study was to analyze the incidence, clinical characteristics, and outcome of inborn SEGA in a large cohort of TSC patients who were followed at the Children’s Memorial Health Institute, Warsaw.

## Material and methods

The study was approved by The Children’s Memorial Health Institute Ethics Committee. The records of TSC patients, who had diagnosis of SEGA prenatally or in the first 3 months of age, were retrospectively reviewed. The inclusion criteria were as follows: diagnosis of SEGA and clinically definite TSC based on Roach’s criteria [5]. SEGA was diagnosed when a tumor was characterized by: location near the foramen of Monro, diameter ≥1 cm, gadolinium enhancement on neuroimaging, and any documented growth, or hydrocephalus present on baseline neuroimaging. Patients with tumors exceeding 1 cm in diameter, but not growing or producing hydrocephalus in the first 3 months of life, were not included in the study.

The analyzed data included the following: patient demographics; mutational analysis results; if available, the presenting symptoms; size of the tumor; treatment applied; any adverse events; and results of follow-up neurological examination and neuroimaging studies.

## Results

In a cohort of 452 TSC patients followed at The Children’s Memorial Health Institute, Warsaw, Poland, 10 (2.2 %) children were diagnosed with SEGA in the first 3 months of life.

Five patients (1.1 % of the whole cohort, 50 % of patients with inborn SEGA) presented with hydrocephalus at baseline, and in all 10 patients, significant tumor growth was observed in the first 3 to 6 months of life. There were eight boys and two girls in this group. Two patients presented clinical symptoms attributed to brain tumor: early focal seizures (one case) or hemiparesis (one case). Table 1 presents clinical data of the patients.

**Table 1:** Characteristics of TSC patents with congenital SEGA

No	Gender	Mutation	Age at baseline neuroimaging	Baseline SEGA size (max diameter) (mm)	Clinical symptoms of SEGA	Hydrocephalus at baseline	SEGA growth	Treatment applied	Follow-up
1	M	UKN	1 week	50	Early seizures	Yes	Yes (to 60 mm at 6 months)	Shunt at the age of 6 weeks and total surgery at 6 months of age.	18 months: no tumor regrowth, persistent hemiparesis
2	M	TSC2/PKD1	Prenatally	40	No	Yes	Yes, to 50 mm at 6 weeks	Subtotal surgery and shunt at 6 weeks. At the age of 10 years, everolimus treatment started	14 years: Persistent hemiparesis, tumor regrowth requiring several reoperations. Significant improvement after everolimus treatment
3	M	TSC2	Prenatally	22	No	Yes	Yes, to 30 mm at 8 months	Shunt at the age of 1 month and total surgery at the age of 8 months	5 years: no tumor regrowth, at the age of 18 months contralateral SEGA developed. Second surgery at the age of 3.5 years
4	M	UKN	1 week	13	No	No	Yes, to 16 mm at the age of 3 months	No treatment: brain ultrasound every month and brain MRI every 6 months	6 months: stable tumor size
5	M	TSC2	2 weeks	10	No	No	Yes: to 13 mm at the age of 2 months	No treatment: brain ultrasound every month and brain MRI every 6 months	4 months: stable tumor size
6	F	TSC2	8 weeks	38	Hemiparesis	Yes	Yes: to 42 mm at the age of 4 months	Total surgery and shunt at the age of 4 months	15 years: no tumor regrowth, improvement in hemiparesis, but at the age of 12 years development of contralateral SEGA. Everolimus treatment introduced.
7	M	TSC2/PKD1	7 weeks	12	No	No	Yes: to 18 mm at the age of 3 months	Everolimus treatment implemented at the age of 12 months	4 years: tumor size decrease by 50 %
8	M	TSC2/PKD1	4 weeks	11	No	No	Yes: to 15 mm at the age of 3 months	Total surgery and shunt at the age of 18 months (SEGA size 18 mm at this moment)	12 years: no tumor regrowth, but at the age of 8 years contralateral SEGA developed. Second SEGA surgery at the age of 10 years.
9	F	TSC2	4 weeks	10	No	No	Yes: to 12 mm at the age of 2 months and 15 mm at the age of 6 months	Total surgery and shunt at the age of 5 years (acute hydrocephalus, SEGA size 40 mm at this moment)	15 years: no tumor regrowth, severe persistent hemiparesis, at the age of 12 years contralateral SEGA developed. Everolimus treatment introduced.
10	M	TSC2	8 weeks	12	No	Yes	Yes: to 25 mm at the age of 6 months	Total surgery and shunt at the age of 6 years, (acute hydrocephalus, SEGA size 60 mm at this moment)	10 years: no SEGA regrowth, persistent visual loss and hemiparesis, at the age of 9 years contralateral SEGA developed. Second SEGA surgery at the age of 9 years

Eight patients had mutational analysis performed and in all of them, mutation in *TSC2* gene was identified. Three of these mutations (37.5 %) were deletions disrupting not only *TSC2* gene but also adjacent *PKD1* gene, causing polycystic kidney disease in these patients.

**Fig. 1:**
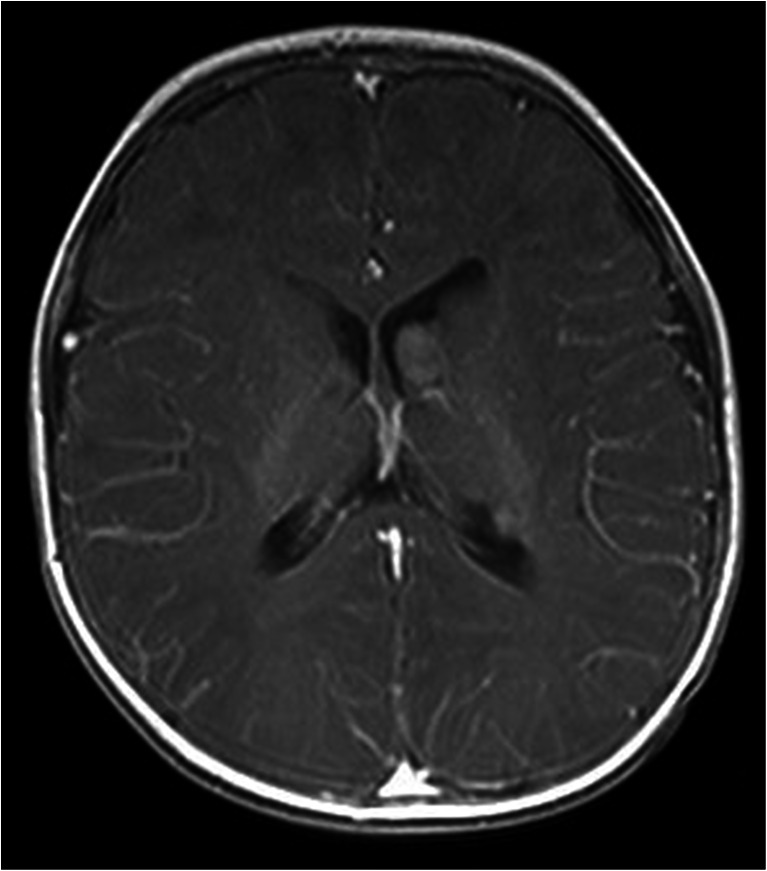
Brain MRI showing SEGA located near the right foramen of Monro and causing hydrocephalus in a 1-week-old patient with TSC

Mean maximum SEGA diameter at baseline was 21.8 mm. Figure 1 presents a patient with 13-mm SEGA in the first week of life. Mean SEGA growth rate observed postnatally was 2.78 mm per month. Mean SEGA size increased by 5.43 mm per month in patients with *TSC2/PKD1* mutations and by 1.76 and 1.35 mm in patients with other *TSC2* mutations and in genetically not tested patients, respectively. The differences were not statistically significant.

Seven patients underwent SEGA surgery. Five patients had surgery during infancy (two at the age of 6 weeks, one at the age of 4 months, one at the age of 8 months, and one at the age of 18 months). These patients were followed by brain ultrasound performed monthly and brain MRI performed every 3–6 months, and SEGA surgery was done when enlargement of ventricles was seen. In all of them, shunt was applied either before (in two patients) or concomitantly (three patients) with tumor resection.

Two patients had SEGA surgery at the age of 5 and 6 years, respectively, both presented with acute hydrocephalus at that time. In both patients, shunt was placed concomitantly with brain tumor resection. In all operated patients, histopathological diagnosis of SEGA was confirmed.

SEGA was removed totally in six out of seven operated patients. In all of them, no tumor regrowth was observed during follow-up of 144.3 months (range 18–180 months). One patient, in whom SEGA was removed subtotally, required several additional surgeries for regrowing tumor and eventually mTOR inhibitor was introduced with significant improvement.

Three patients had no surgery. Two of them are still infants (6 and 4 months of age) and are followed with serial neuroimaging studies. In one patient, mTOR inhibitor (everolimus) was introduced at the age of 12 months. In this case, SEGA size decreased by 50 % during 4-year follow-up and the patient is still on treatment.

Surgery-related complications were observed in four patients (57.1 % of operated cases). Three surgeries were uneventful. The complications included the following: persistent hemiparesis (four patients) and visual impairment (one case).

Five of operated patients developed contralateral SEGA in 18 months to 12 years after the first surgery. Three of them required second surgery, and two were given medical treatment with everolimus.

Altogether, four patients were treated with everolimus: either as primary treatment (one patient) or because of tumor regrowth (one case) or because of new SEGA development (two patients). No new tumor growth was observed in any of patients treated with everolimus.

## Discussion

SEGAs are the most common brain tumors occurring in up to 20 % of the TSC patients, usually in the first two decades of life [6, 23, 35]. They are typically located on the surface of the lateral ventricle of the brain, and thus, while growing, they extend into the lateral ventricle and can obstruct the foramen of Monro and flow of CSF, causing hydrocephalus [8, 10]. SEGAs usually grow slowly, and the mean age they present clinical symptoms or cause hydrocephalus is 9.7 years [16, 26, 29, 37]. However, in rare cases, rapid significant increase in tumor size was observed, especially in the youngest children [12, 17]. There are also reports on SEGA growth in newborns and fetuses [13, 31, 33]. Histopathologically, some SEGAs in very young children may present features of malignancy, which are not typical for this type of tumor [12].

This is the first analysis of incidence, clinical features, and outcome of neonatal SEGA in TSC individuals. In our large cohort of TSC patients, congenital SEGA was present in 10 cases (2.2 %). It is important to note that in this report, inborn SEGA was recognized only in patients in whom either hydrocephalus associated with SEGA or significant tumor growth within the first 3 months of age was documented. It cannot be excluded that some cases of congenital SEGA, which were asymptomatic at birth and grew slowly, were overlooked in early infancy and diagnosed later in life. In our group of patients with early diagnosed SEGA, two had surgery postponed to the age of 5 and 6 years, respectively. They required surgery because of huge tumors and acute hydrocephalus, but they had been asymptomatic up to this moment. One of those patients was lost from follow-up for 4 years, and the parents of the other one did not give consent for surgery as long as the patient had no SEGA symptoms. Considering those cases, the possibility that more cases of congenital SEGAs are not being recognized seems to be likely.

Although rare, congenital SEGAs present significant clinical problem. Surgical treatment of brain tumors in young infants remains a challenging endeavor, and even in benign tumors, mortality and morbidity in these patients is higher than that in other pediatric age groups [21]. In our study, there were no deaths, but 57 % of operated patients experienced significant neurological deficits associated with surgery. This is consistent with our previous reports, showing that age <3 years is a risk factor for SEGA surgery complications [16].

Five of our operated patients had SEGA resection and shunt implantations performed during one surgery, and in two patients, shunt was placed earlier (at the age of 4 and 6 weeks), and SEGA surgery was postponed to the age of 8 and 6 months, respectively. Both approaches were associated with risk of surgery-related sequelae, although it should be noted that the patient who had shunt prior to SEGA surgery at the age of 6 months was the one to have the largest tumor in the cohort (60 mm in diameter) and SEGA size exceeding 4 cm is an important risk factor for surgery complications [4, 16, 30]. Therefore, it cannot be excluded that in some cases, earlier shunt implantation and postponed SEGA resection might be beneficial.

Six out of seven operated patients in this cohort had total SEGA surgery and in one, partial resection was performed. Accordingly, to other reports [2, 16, 30], no tumor regrowth was observed after total SEGA surgery, but several additional surgeries were performed in a patient after partial resection. It should be noted, however, that in five patients, contralateral SEGA developed over the next 1.5 to 12 years. This finding suggests that patients who had one SEGA might require more frequent neuroimaging studies than patients who had never had such tumor.

In our cohort of newborns with SEGA, *TSC2* mutations, including *TSC2* large deletions affecting *PKD1* gene, were identified in all of patients who had mutational analysis done. No patient with inborn SEGA had *TSC1* mutation. It is consistent with our previous studies showing that SEGA develop significantly more earlier in individuals with *TSC2* mutations than in TSC1 mutation patients [16]. Moreover, this study shows that patients with large genomic mutations affecting both *TSC2* and *PKD1* genes are at significantly higher risk of early development of SEGA than patients with other mutations in TSC2 genes. TSC2/PKD1 mutations account for 2–3 % of all TSC cases [6, 34], but in our cohort, these mutations were found in 30 % of patients. Our results indicate that patients with polycystic kidneys and TSC should be screened for SEGA from birth.

In this study, SEGAs in patients with *PKD1/TSC2* mutations tended to grow more rapidly than in patients with other *TSC2* mutations and patients with unknown mutations. The differences between the groups were not significant (*p* = 0.19 and 0.21, respectively) but it was likely associated with small groups. Nevertheless, patients with large *PKD1/TSC2* mutations and SEGAs should be followed with frequent neuroimaging studies.

Recently, an mTOR inhibitor, everolimus, was approved by EMA and FDA for medical treatment of SEGA associated with TSC [9, 14, 18]. The data on safety of everolimus in infants and young children is very limited, but in a small cohort of TSC children below 3 years of age, such treatment was reported to be safe and effective [17]. In this study, one patient had everolimus introduced as a primary treatment for SEGA and one as a adjuvant therapy after partial SEGA resection, and two additional patients everolimus was started because of the development of contralateral tumor. All these patients are continuing treatment. The patient who is given everolimus for primary treatment of SEGA at the age of 12 months has been being followed for 4 years. SEGA size in this child decreased by 50 %. None of the patients treated with everolimus developed new tumor, but it should be noted that in two of them, everolimus was introduced because of contralateral tumor growth. The possibility that medical treatment with mTOR inhibitor might prevent or reduce the risk of SEGA development requires further studies.

In conclusion, we showed that congenital SEGA develops 2.2 % of TSC patients. Patients with *TSC2* mutations, and especially with *TSC2/PKD1* mutations, are more prone to develop SEGA earlier in childhood and should be screened for SEGA from birth. In young infants with SEGA, both surgery and mTOR inhibitor should be considered as a treatment option.

## Abbreviations

SEGA: Subependymal giant cell astrocytoma
TSC: Tuberous sclerosis complex
